# Do Striped Hyenas Have a Sweet Tooth? First Evidence of Honey Consumption by a Hyaenid

**DOI:** 10.1002/ece3.72485

**Published:** 2025-11-16

**Authors:** Francisca A. S. Virtuoso, Robert Milia Kaai, Yorick Liefting, Femke Broekhuis, Rebekah Karimi, James Kisau, Loontasati Lolari, Silole Tumaina, Soloomon Lekai, Gibson Lepilal, Richard Silamui, Oningoi Partayo, Purity Selelo, Joseph Sayiore, Noah Tingai, Wilson Ntitika, Duncan Korongoro, Elvis Nemagai, Jesephat Njue, Kelvin Tajeu, Taiko Nanguyien, Patric Namusu, Richard Stratton Hatfield

**Affiliations:** ^1^ Environmental Sciences Group Wageningen University and Research Wageningen the Netherlands; ^2^ The Kenya Bird of Prey Trust Naivasha Kenya; ^3^ The Kwenia Vultures Sanctuary Trust Kajiado County Kenya; ^4^ The Bird of Prey Trust Borssele the Netherlands

**Keywords:** alternative foods, camera trap, carnivores, diet, honey comb, Kenya, striped hyena

## Abstract

Dietary flexibility in carnivores remains an underexplored aspect of their ecology, particularly the consumption of non‐meat foods. While striped hyenas ( *Hyaena hyaena* ) are primarily scavengers, they are known to occasionally consume plant material, insects and other alternative food sources. Here, we present the first documented evidence of honey consumption by a hyaenid species. During a community‐led camera trap survey in October 2024 at the Kwenia Vulture Sanctuary, Kenya, an adult striped hyena was photographed carrying a large honeycomb in its mouth. This observation suggests ingestion of honey, beeswax and bee larvae, providing nutritional, medicinal or hydration benefits. Our finding expands current knowledge of hyena dietary plasticity and highlights the limitations of conventional diet analysis methods in detecting rare food items such as honey. It underscores the value of complementary observational tools, such as camera, trapping in capturing elusive or unexpected dietary behaviours.

## Graphical abstract

Dietary flexibility in carnivores is often overlooked, particularly regarding the consumption of non‐meat food items. We report the first documented case of honey consumption by a hyaenid: A striped hyena ( *Hyaena hyaena* ) was photographed carrying a large honeycomb during a community‐led camera trap survey in Kenya. This observation highlights the species' dietary plasticity and underscores the value of camera trapping for detecting rare or unexpected feeding behaviours.

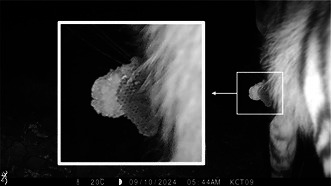

## 1 Introduction

Diet is fundamental to the survival and fitness of species, shaping their physical and behavioural adaptations, as well as the ecological niche they occupy (Stephens 2019). For carnivores, adapted to consume animal flesh, meat provides a highly efficient energy source due to its similar chemical composition to the consumer (Day et al. 2022). Despite this, even obligate carnivores occasionally consume plant material or other non‐meat foods (Yoshimura et al. 2021). This behaviour, while unusual, has been observed in many carnivorous species, though the reasons remain unclear (Montalvo et al. 2020; Sueda et al. 2008; Yoshimura et al. 2021).

Hyaenids are a small yet diverse clade of carnivores comprised of five extant species whose diverse diets represent the adaptability and dietary flexibility of this guild (Wilkinson et al. 2024). More specifically, the most broadly distributed species of hyena, the striped hyena ( *Hyaena hyaena* ), frequently consumes a broad range of foods dominated by scavenged meat (Alam and Khan 2015; Bhandari et al. 2020; Mwebi et al. 2024). They have also been found to supplement their diet with alternative food sources that include fruits and vegetables; grasses and leaf material; insects; and eggs (Alam and Khan 2015; Kuhn 2005).

An alternative food source not currently known as part of the diet of striped hyena and hyaenids broadly, is honey. Honey consumption is a rarely reported alternative food for carnivores. To our knowledge, the only Carnivora species reported to consume honey includes the pine marten (*Martes martes*; Monterroso et al. 2016), the honey badger ( *Mellivora capensis* ; Begg et al. 2003) and the brown bear ( *Ursus arctos* ; Lalleroni et al. 2017).

Here, we contribute to the list of honey intake in the carnivore guild, by presenting the first evidence of honey consumption by striped hyena ( *Hyaena hyaena* ).

## 2 Field Record

**FIGURE 1:**
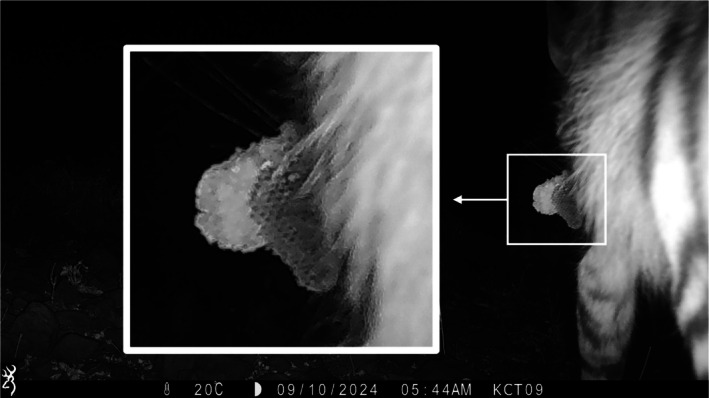
Camera trap image of an adult striped hyena at the Kwenia Vulture Sanctuary, Kajiado County, Kenya, carrying a honeycomb, likely for consumption. This photo was taken on 09 October 2024 at 5:44 am at −1.83646849, 36.49852795 with a Browning camera trap.

**FIGURE 2:**
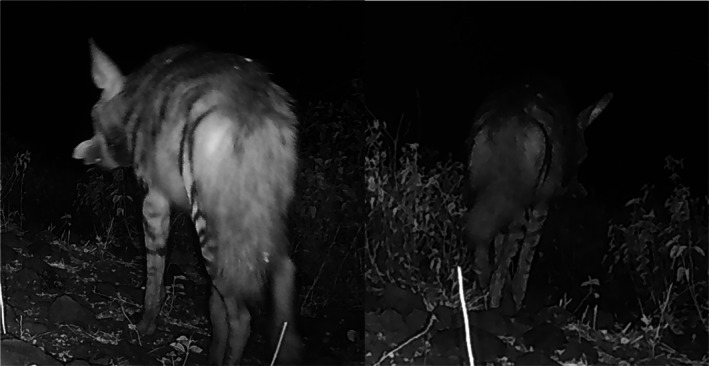
Two camera trap images showing an adult striped hyena at the Kwenia Vulture Sanctuary, Kajiado County, Kenya, carrying a large piece of honeycomb, visibly protruding from both sides of its mouth. These photos were taken on 09 October 2024 at 5:44 am at −1.83646849, 36.49852795 with a Browning camera trap.

We present a series of camera trap images capturing an adult striped hyena carrying a honeycomb, which we interpret as evidence of honey consumption by the individual or its offspring (Figure 1). The honeycomb is of significant size as it can be seen protruding from both sides of the striped hyena's mouth (Figure 2).

**FIGURE 3:**
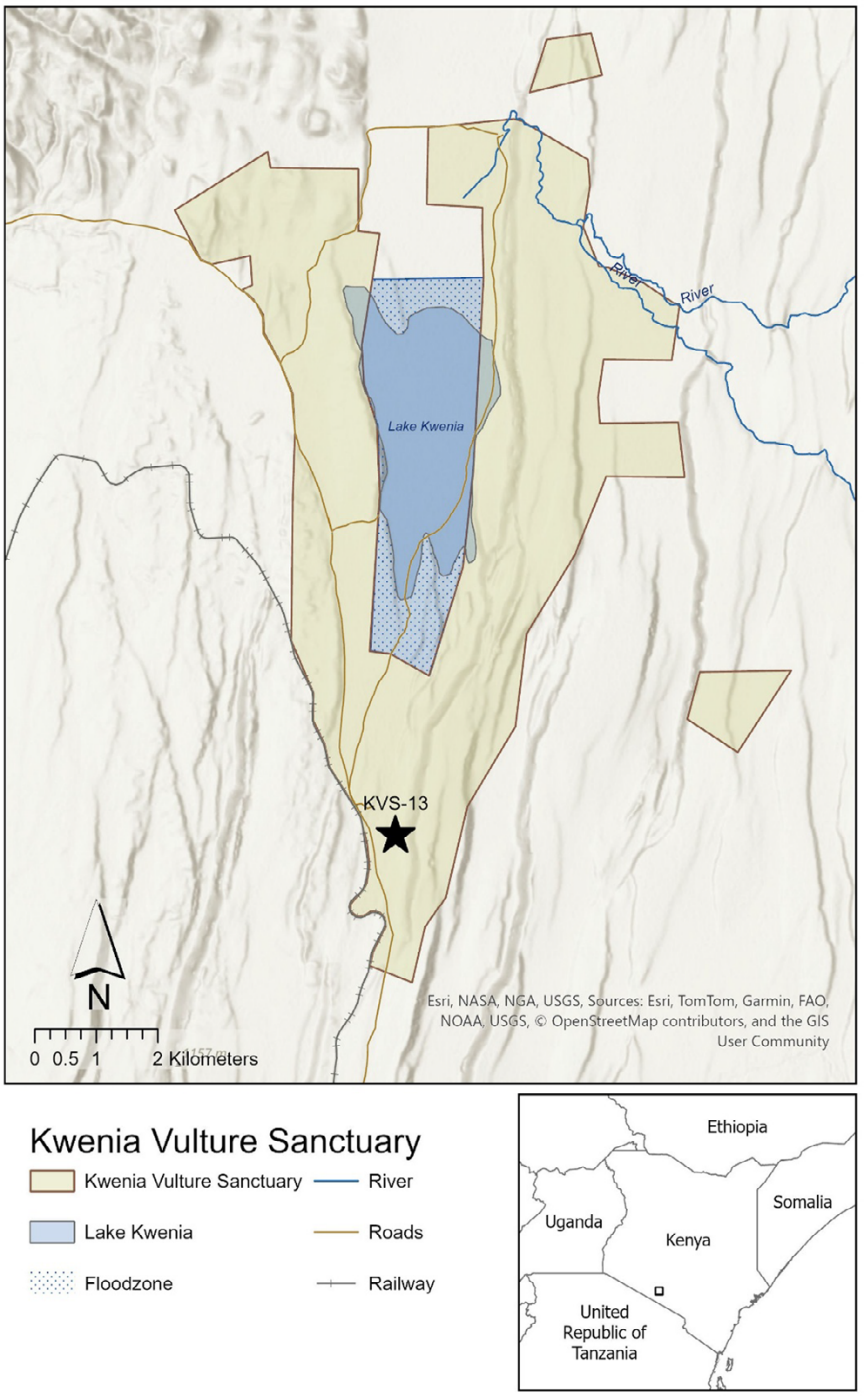
Map of the Kwenia Vulture Sanctuary, highlighting location KVS‐13 (−1.83646849, 36.49852795), where the images in Figures 1 and 2 were captured.

This observation was made on 09 October 2024 at 5:44 am as part of a larger ongoing community conservation camera trap survey at the ~5140‐ha Kwenia Vulture Sanctuary in Kajiado County, Kenya (−1.809365, 36.498251; Figure 3). The images were taken by a Browning 2021 Recon Force Elite HP4 camera trap placed 30 cm above the ground, to maximise small‐ to medium‐sized mesocarnivore detectability.

The study area, Kwenia, is part of the Somali–Maasai biome, characterised by arid Acacia–Commiphora savannah. It is centred around a large seasonal lake and an extensive cliff line, which supports the largest known breeding colony of Rüppell's vulture (*Gyps rueppelli*) in southern Kenya. The region hosts a diverse mammalian community, including a robust population of striped hyena alongside other carnivores such as spotted hyena ( *Crocuta crocuta* ), leopard ( *Panthera pardus* ), cheetah ( *Acinonyx jubatus* ), African wild dog ( *Lycaon pictus* ), caracal ( *Caracal caracal* ), and serval ( *Leptailurus serval* ). This predator guild is sustained by a varied herbivore population, including Grant's gazelle ( *Nanger granti* ), Kirk's dik‐dik ( *Madoqua kirkii* ), lesser kudu ( *Tragelaphus imberbis* ), gerenuk ( *Litocranius walleri* ) and Maasai giraffe ( *Giraffa tippelskirchi* ). Depredation of livestock (goat, sheep, cattle, donkeys) by predators is common as well. Kwenia Vulture Sanctuary is owned and managed by the indigenous Maasai pastoralist community, who also graze their livestock in and around the sanctuary. Traditionally honey hunting has been common practice at Kwenia, which is now rapidly evolving towards beekeeping. Products derived from this practice, including honey, honeycombs, and bee larvae, are considered valued delicacies and are commonly shared within the community.

## 3 Discussion

To our knowledge, this is the first evidence of honey consumption by a hyaenid. Striped hyenas access the bulk of their diet (meat) mainly by scavenging and supplement it with alternative food sources, such as fruits or insects, in resource‐scarce environments (Alam and Khan 2015; Mwebi et al. 2024). Consumption of such foods has been hypothesised to be due to compensation for water scarcity, self‐medication against pathogens, aid digestion or simply due to added nutritional value (Day et al. 2022; Yoshimura et al. 2021).

Importantly, in our record, the striped hyena was carrying a honeycomb, which does not only contain honey but also beeswax and potentially bee larvae. The combination of these components may contribute to all these supplementary benefits. Honey is comprised mainly of water and sugars, making it a highly nutritious food source (Ajibola et al. 2012), providing energy and potentially serving as a substitute for water in scarce environments. Beeswax has antimicrobial and anti‐inflammatory properties, and its consumption could possibly be a form of self‐medication (Fratini et al. 2016). Finally, insect larvae have previously been recorded to be consumed by several carnivore species, as it is a highly nutritional source of protein (Finke 2002). Aside from these physiological benefits, perhaps there is simply an element of preference for something sweet.

Physiological studies have shown that several carnivores, although not all, have lost the taste receptor for sweetness (Jiang et al. 2012; Li and Zhang 2014). For example, although spotted hyenas have lost the ability to taste sweet foods, other hyena species, such as aardwolves, still have it (Jiang et al. 2012). Captive striped hyenas in the DierenPark ZieZoo in the Netherlands have a clear preference for consuming sugar‐rich fruits, such as bananas, pears and grapes (Patrick Rutjes, pers. comm, 2025), perhaps having developed a preference for sweet foods. In our study area, communities report that striped hyenas raid and consume watermelons from agricultural fields. It may be that striped hyenas have not lost this taste receptor, and that could be an additional reason for honey consumption. Other carnivores, such as bears, that have been reported to consume honey, also still have this receptor (Jiang et al. 2012).

Honey consumption in carnivores broadly is very difficult to detect. Diet studies usually focus on meat consumption, either by extracting prey hair from scats or by targeting vertebrate DNA. Unless the animal is physically observed eating or DNA primers targeting honeybees are used, it is simply not possible to detect honey consumption in traditionally used dietary analysis methods (Alam and Khan 2015; Monterroso et al. 2016; Mwebi et al. 2024). Currently, there have been no studies on striped hyena diet that have explored the possibility of them consuming honey. The closest finding has been insect remains in scats, which were not identified to the species level (Alam and Khan 2015). These biases in carnivore diet studies present a unique challenge.

Our findings suggest that honey, although rarely documented in carnivore diets, may be more common than previously thought. Given the limitations in current diet analysis methods and study focus, other instances may simply go undetected. For example, several studies have shown the consumption of bee larvae by carnivores, such as the honey badger (Begg et al. 2003), but this may be complemented with honey consumption, which is more difficult to detect than insects in diet studies. Fortunately, striped hyenas are well known for carrying food back to den sites for either self‐consumption or the provisioning of offspring (Hadad et al. 2023), which provides the opportunity to detect and understand the rare consumption of non‐meat components, which can be detected by non‐dietary focused methods, such as camera trapping.

This unexpected dietary choice by striped hyena aligns with sporadic reports of carnivores incorporating unconventional food sources into their diet (Montalvo et al. 2020; Sueda et al. 2008; Yoshimura et al. 2021). This observation adds to our understanding of hyena dietary plasticity, underscoring the species' resilience and adaptability in varying environmental conditions.

## Author Contributions

**Francisca A. S. Virtuoso:** conceptualization (lead), visualization (equal), writing – original draft (lead). **Robert Milia Kaai:** methodology (equal), project administration (equal), resources (equal), writing – review and editing (equal). **Yorick Liefting:** conceptualization (equal), data curation (lead), methodology (equal), writing – review and editing (equal). **Femke Broekhuis:** conceptualization (equal), data curation (equal), methodology (equal), writing – review and editing (equal). **Rebekah Karimi:** methodology (equal), project administration (equal), resources (equal), writing – review and editing (equal). **James Kisau:** methodology (equal), resources (equal), writing – review and editing (equal). **Loontasati Lolari:** methodology (equal), resources (equal), writing – review and editing (equal). **Silole Tumaina:** methodology (equal), resources (equal), writing – review and editing (equal). **Soloomon Lekai:** methodology (equal), resources (equal), writing – review and editing (equal). **Gibson Lepilal:** methodology (equal), resources (equal), writing – review and editing (equal). **Richard Silamui:** methodology (equal), resources (equal), writing – review and editing (equal). **Oningoi Partayo:** methodology (equal), resources (equal), writing – review and editing (equal). **Purity Selelo:** methodology (equal), resources (equal), writing – review and editing (equal). **Joseph Sayiore:** methodology (equal), resources (equal), writing – review and editing (equal). **Noah Tingai:** methodology (equal), resources (equal), writing – review and editing (equal). **Wilson Ntitika:** methodology (equal), resources (equal), writing – review and editing (equal). **Duncan Korongoro:** methodology (equal), resources (equal), writing – review and editing (equal). **Elvis Nemagai:** methodology (equal), resources (equal), writing – review and editing (equal). **Jesephat Njue:** methodology (equal), resources (equal), writing – review and editing (equal). **Kelvin Tajeu:** methodology (equal), resources (equal), writing – review and editing (equal). **Taiko Nanguyien:** methodology (equal), resources (equal), writing – review and editing (equal). **Patric Namusu:** methodology (equal), resources (equal), writing – review and editing (equal). **Richard Stratton Hatfield:** conceptualization (equal), methodology (equal), project administration (equal), visualization (equal), writing – original draft (equal).

## Ethics Statement

The authors have nothing to report.

## Conflicts of Interest

The authors declare no conflicts of interest.

## Data Availability

All data are included in this manuscript. There is no additional data or code associated with the research.
